# Molecular characterization and DNA methylation profile of *Libyodrilus violaceous* from oil polluted soil 

**DOI:** 10.22099/mbrc.2019.35242.1449

**Published:** 2020-06

**Authors:** Aemere Ogunlaja, Vikas Sharma, Meenu Ghai, Johnson Lin

**Affiliations:** 1 Department of Biological Sciences, Redeemer’s University, Ede Osun state, Nigeria; 2 School of Life Sciences, Discipline of Microbiology, University of Kwazulu-Natal, Durban, South Africa; 3 Department of Biotechnology Engineering, Ambala college of Engineering and Applied Research, Devsthali, P.O Sambhalkha, Ambala-133101, Haryana, India; 4 School of Life Sciences, Discipline of Genetics, University of Kwazulu-Natal, Durban, South Africa

**Keywords:** Oil spill, Pollution, Natural attenuation, DNA Barcode, Phylogeny

## Abstract

Studies on earthworms using molecular markers are rare in Africa except a handful from South Africa. Reports on *Libyodrilus violaceous*, an earthworm found in West Africa are available including their metal tolerance and bioaccumulation capacity but their molecular characterization and ecotoxicology studies are scarce. In this study, triplicate *L. violaceous* specimens were collected from four locations within a petroleum polluted site and one in a control site, ≃1Km away from point of spill. DNA was extracted and 18S rRNA and 16S rRNA genes were amplified and sequenced. DNA methylation of their 18S rRNA gene was determined using Methylation specific PCR (MSP) method. Phylogenetic trees generated for 18S rRNA and 16S rRNA genes grouped *L. violaceous *within the Eudrilidae family concurrent with its conventional grouping and MSP results indicate no methylation in *L. violaceous *population from this site.

## INTRODUCTION

Earthworms are ecologically important soil fauna hence their role as terrestrial bioindicators. The estimated earthworm species worldwide was approximated as 3000 in 2007 [1] and 3700 species in 2008 [2]. With molecular methods, there are estimated; 67 species in Greece [3], 90 species in Balkan Peninsula [4], 28 species in Guadeloupe [5] and 212 species in Vietnam [6]. Figures on indigenous African earthworm species are lacking and their sequences are few in repositories. 

The conventional earthworms’ taxonomy is labour-intensive, takes time and requires expertise. Some features of earthworms used for earthworm taxonomy at family level are now unreliable [1]. Molecular approach for characterization and earthworm ecological studies outweighs that of conventional methods [7-9]. Others argue that there are earthworm identification mismatch in both methods. Depending on one DNA region for molecular identification could be inaccurate as regions like mitochondria are known for their paraphyly and polyphyly [10-12]. More studies to investigate and corroborate previous classifications are therefore necessary.

DNA barcoding [13] using 18S rRNA and 16S rRNA genes and DNA methylation profiling (MSP) are the molecular methods used in classification and ecotoxicology in this study. 18S rRNA gene is recommended for taxonomic groupings of eukaryotes [14] while 16SrRNA gene are commonly used in classifying prokaryotes [15] and such sequences are found in the GenBank [16, 17]. Methylation occurs in the DNA as organisms respond to environmental stress like heavy metal pollution [18, 19] resulting in gene expressions or repressions [20]. Kille, showed DNA methylation among earthworm species in arsenic polluted site [21]. Gross also showed the variability of the 18S rRNA locus of three fish species seen in their chromosomal rearrangement [22] and Li established cysteine methylation of 18SrDNA gene in Arabidopsis from Nickel polluted soil [23].

The most common African earthworm family is Eudrilidae [24], endemic to tropical and sub-tropical soils, a taxa with 45 genera and 350 species [25] but only 34 nucleotide sequences and 11 protein sequences of the Eudrilidae family are found in NCBI /DDBJ/EMBL/GenBank. Beddard [26] first described *Libyodrilus violaceous *as a West African earthworm species isolated from Lagos, conventionally classified under the family Eudrilidae [24], subfamily Polytoreutinae and genus *Libyodrilus*. They have ‘acorn-shaped’ enlarged central chamber [27], ventrally located tubular structured prostrates and prostratic pores with male pore at the 17/18 intersegmental furrow. Their spermatheca also combines with ovarian duct as an organ at segment 14. Unlike other members of the family, they lack dorsal pores and nephridiopore [26]. Other genera in the family Eudrillidae include *Hyperiodrilus* and *Polytoreutus*.

*L. violaceous *are found in muddy soils abundantly during the wet seasons [28]. They appear purplish and luminescent occurring mostly twined together forming rope-like bundles [26, 29-32]. The species is found in Cameroon and distributed from the middle belt spanning down the southern part of Nigeria [32]. The importance of this earthworm species are highlighted in investigations including physiology [33], ecology [28], nutrition [34], ecotoxicology [35-39] especially its ability to tolerate polluted soils [28, 38] and metal bioaccumulation potential [40]. Except from a few molecular reports on *L. violaceous *[38, 40], there is dearth of information in its molecular characterization and ecotoxicology. This study involved molecular characteriza-tion of *L. violaceous* using 16SrRNA and 18SrRNA genes and determination of DNA methyla-tion in *L. violaceous *specimens from a site eight years post impact of oil spills in a pipeline vandalized area in Lagos State, Nigeria [28].

## MATERIALS AND METHODS

**Molecular characterization Earthworm Collection: **Fifteen *L. violaceous* samples were collected from the oil polluted site at Agaye, Ije-Ododo, Alimosho LGA of Lagos State Nigeria (Lat. 06^o^ 29'N and Long. 03^o^ 15' E). Triplicate samples were collected from five different points ≃100m apart: LV1 (N 06^o^ 29. 40', E003^o^ 15. 23'), LV2 (N 06^o^29.41', E003^o^ 15.20'), LV3 (N 06^o^ 29.42', E003^o^15.24') and LV4 (N 06^o^29.44', E003^o^ 15.19') within the site and LV5 (N 06^o^ 30.00', E003^o^15.25') ≃1km away from point of spill as control. Earthworms were collected by hand from 0 – 15cm depth of soil surface and allowed to depurate for 24hrs in the laboratory. 3-5mm of the anterior end of each earthworm was cut and remaining gut content removed; this portion was preserved in RNA shield (ZYMO RESEARCH) for further molecular procedures. Earthworm specimens were identified conventionally by Prof.Owa, an earthworm taxonomist from the Zoology Department, Osun State University, Nigeria [28]. 

**DNA Extraction, PCR amplification and Sequencing of 18S rRNA and 16S rRNA genes: **≤25mg anterior portion of each sample was cut and grinded in Liquid Nitrogen. DNA extraction was done using *Quick*-DNA^TM ^Universal kit (ZYMO RESEARCH) following the manufacturer’s procedure. Isolated DNA were amplified with final volume of 20 µl using 2 µl (10mM) of primers: 16SrRNA; F: CGA CTG TTT AAC AAA AAC AT (ewA) and R: CGC GGT CTG AAC TCA GCT CAT G (ewF) (≃ 520 bp) [41] and 18SrRNA; F: CAG CAG CCG CGG TAA TTC C (F-566), R: CCC GTG TTG AGT CAA ATT AAG C (R-1200) (≃650 bp) [42]. Also 2µl DNA template, 10µl PCR Master mix (Phusion High-Fidelity) and 4 µl nuclease H_2_O. The PCR conditions were as follows; one cycle of 10 mins at 95^o^C 34 cycles of 30s at 95^o^C 40s at 48^o^C and 50^o^C for 16S rRNA and 18S rRNA respectively, 1 min of 72^o^C; then 5 mins of 72^o^C. Gel electrophoresis using 1% (w/v) Agarose gel stained with ethidiumbromide (10mg/ml) was done to visualize the PCR amplicons. Representative PCR amplicons were sequenced using Sanger sequencer (Central analytical facility, Stellenbosch University).

**Phylogenetic analyses** **: **The 18S rRNA and 16S rRNA nucleotide sequence reads were compared with reference sequences obtained from NCBI GenBank using the Basic Local Alignment Search *Tool* (*BLAST*). Sequence alignments were done with MAFFT program using the L-INS-i algorithm (maximum likelihood) (ultrafast bootstrap with 1000 replicates). The Phylogenetic trees were generated with the command-line version of the IQ-tree software version 1.5.5 [43- 46].

**Methylation specific PCR (MSP): **DNA extracts from the specimens (LV1 - LV5) were subjected to bisulphite conversion using EZ DNA Methylation lightening kit (Zymo research, Irvine, CA USA) following the manufacturer’s instruction. CpG islands with high CpG density were identified in 18SrRNA nucleotide of *L. violaceous *and two sets of primers each (Forward and reverse primers) specific for methylated and unmethylated cytosine targeting the CpG islands were designed using Meth Primer 2 design software. Each PCR amplification were done using 50 µl mixture with 25 µl of GoTaqHot Start Green Master mix (Promega, coop), 0.75µl forward and 0.75µl reverse primers both specific for methylated and unmethylated, 2µl of bisulphite converted DNA template and 21.5 µl of nuclease free water. The PCR condition was one cycle of 10mins at 95^o^C; 35 cycles of 30s at 95^o^C,40s at 44^o^C and 42^o^C for methylated and unmethylated respectively, 1min of 72^o^C;then 7 mins of 72^o^C. PCR products were resolved by electrophoresis on 2% (w/v) ethidium bromide stained agarose gel.

## RESULTS

16S rRNA and 18S rRNA genes were successfully amplified and viewed at the expected sizes (**≃**520bp and **≃**650bp respectively) [41-42]. The partial nucleotide sequences of the two genes of the representative *L.violaceous *specimens are deposited in DDBJ/EMBL/GenBank with accession numbers KY114795.1 (18SrRNA) and KY114796.1 (16S rRNA). The 18S rRNA nucleotide sequence of *L.violaceous* shows this earthworm species had close genetic relationship with *Eudriloides* sp. *Polytorentus finni *and *Hyperiodrilus* sp. (Table 1). Similarly, the 16SrRNA nucleotide sequence BLAST indicates close identities with *Eudriloides* sp.; *Hyperiodrilus* sp; *Metaphire sieboldi*; *Dichogaster *sp and* P. finni *(Table 2)*.*

**Table 1:** *L. violaceous* 18S rRNA nucleotide sequence BLAST percentage coverage and identity compared with genetically closely related earthworm species

**Organisms**	**Accession number**	**Percentage Identity**	**Percentage coverage**
*L. violaceous*	KY114795.1	100%	100%
* Eudriloides* sp. Ke	HQ728924.1	99%	100%
* Polytorentus finni*	HQ728926.1	98%	100%
* Hyperiodrilus* sp. Gh	HQ728925.1	98%	100%

**Figure 1:**
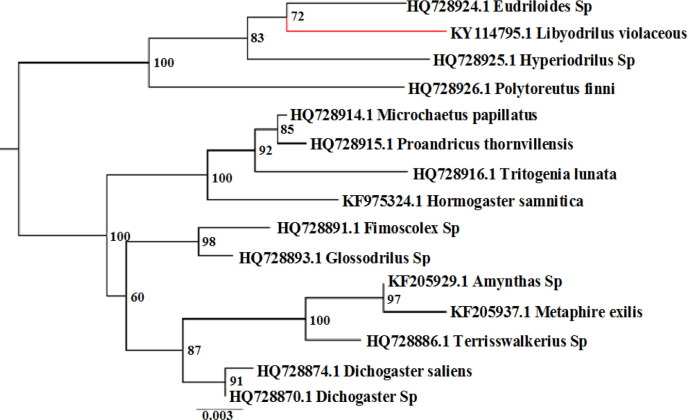
Phylogenetic tree of 18S rRNA nucleotide sequence of *L. violaceous *(indicated in red) compared with other earthworms’18SrRNA nucleotide sequences

**Figure 2:**
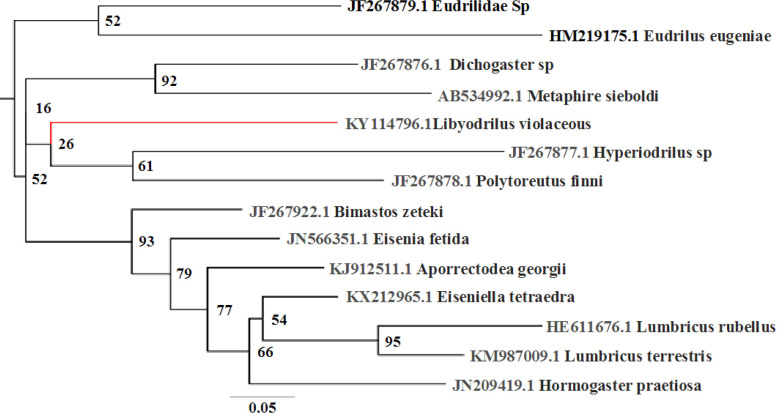
Phylogenetic tree of 16S RNA nucleotide sequence of *L. violaceous *(indicated in red) compared with some earthworms’16S rRNA nucleotide sequences

The molecular classification of *L. violaceous *with 18S rRNA gene is depicted in the phylogenetic tree below (Fig. 1). The tree has two major clusters, the first comprising of the Eudrilidae group with the species; *L. violaceous*, *P. finni*, *Hyperiodrilus* sp. and *Eudriloides *sp. and the second with species of the families Microchaetidae, Tritogeniidae, Hormogastridae, Glossoscolecidae, Megascolecidae, and Acanthodrilidae of the superfamily Megascolecoidae. The molecular classification of *L.violaceous *using16S rRNA gene is depicted in the phylogenetic tree below (Fig. 2). The tree shows two major sister clusters, the first, a clade consisting of *Eudrilidae *sp. and *Eudrilus eugeniae* with a common root. The second sister cluster has three sub-clusters with the first having *Dichogaster *sp. and *Metaphire sieboldi* nested in one clade and the second clade having *L. violaceous, Hyperiodrilus* sp. and* P. finni.* The third subcluster shows the remaining reference earthworm spp. There was no variation in the specimens of *L. violaceous* within the polluted site compared to the control.

**Table 2:** *L. violaceous* 16S rRNA nucleotide sequence BLAST percentage coverage and identity compared with genetically closely related earthworm species

**Organisms **	**Accession number**	**Percentage Identity**	Percentage coverage
***L. violaceous***	KY114796.1	100%	100%
***Eudriloides*** ** sp. Ke**	JF267879.1	82%	97%
***Hyperiodrilus*** ** sp. Gh**	JF267877.1	82%	97%
***Dichogaster *** **sp**	JF267876.1	81%	96%
***Metaphire sieboldi***	AB534992.1	82%	92%
***P. finni***	JF267878.1	80%	72%

## DISCUSSION

The 16S rRNA and 18S rRNA genes were successfully amplified. The percentage coverage and identity of the sequence reads of both genes and phylogenetic trees corroborate the conventional classification of *L. violaceous* as a Eudrillidae. 

The closest relative of *L. violaceous* is *Eudriloides* sp. It originates from Kenya, central Africa. James and Davidson [47] had reported *Eudriloides* sp. as more evolutionary related with *Hyperiodrilous *sp. in the 18S+28S partitioned analysis, Bayesian phylogram. They also reported this species to be equally related to *P. finni *and *Hyperiodrilus africanus *in a 28S gene tree. *P. finni* and *Hyperiodrilus* sp. are of the genus *Polytoreutus* and *Hyperiodrilus*, respectively, of the family Eudrilidae [24]; all four species are seen clustered in one clade in our study (Fig. 1) and is consistent with the conventional grouping of the species [26, 29].

The second cluster of the phylogram (Fig. 1). has two subclusters with the first showing a clade of two species of the family Microchaetidae; *Microchaetus papillatus* and *Proandricus thornvillensis*. Also within this subcluster is *Trigenia lunata* of the family Tritogeniidae formerly of Microchaetidae [47-51] recently allotted its own family Tritogeniidae by Plisko in 2013 [52]. The species *Hormogaster samnitica *is also within this subcluster. Plisko [53] had suggested Hormogastridae having genetic relationship with Tritogeniidae. 

The second subcluster shows a clade of Glossoscolecidae; *Fimoscolex *sp., and *Glossodrilus *sp. [53, 54]; The closeness of Glossoscolecidae to Eudrilidae had earlier been highlighted as they have similar ejaculatory structure [47, 55] and they possess euprostate and paired dorsal calcium carbonate gland in either segment 12 or 13 [47]. The second subcluster also shows a clade of the family Megascolecidaeis;* Amynthus *sp, *Metaphire exilis* and *Terrisswakerius *sp. and another of the Acanthodrilidae family;* Dichogaster saliens* and *Dichogaster *sp. All species in the second subcluster are from the super family Megascolecidae. The phylogenetic tree generated clusters that indicate a common origin of all species being oligochaetes; of the polyphyletic order Crassiclitellata [56-57]. 

The phylogenetic tree for the 16S rRNA gene (fig. 2) shows weak clustering of *Eudrilidae *sp. and *Eudrilus eugeniae* species separate from the other clade of *L. violaceous, Hyperiodrilus* sp. and* P. finni* although they are all of the Eudrilidae family. This could be due to the polyphyletic nature of the Eudrilidae family [47, 51], it could also be because 16S rRNA gene is best for prokaryotic classification [15]. *Dichogaster *sp. and *Metaphire sieboldi *in the second cluster are conventionally of the super family Megascolecidae. The third sub-clade clusters *Aporrectodea georgii*, *Eisenia fetida*, *Bimastos zeteki*, *Eiseniella tetraedra*, *Lumbricus rubellus *and *Lumbricus terristris *(of the family Lumbricidae) with *Hormogaster praetiosa *(of the same super family Lumbricoidea).

The phylogenetic grouping in this study (Fig. 1, 2) supports the conventional classification of *L. violaceous *with the18S rRNA gene as more reliable than the 16S rRNA gene for its molecular taxonomic grouping. 18S rRNA gene is acclaimed as more appropriate for eukaryotic classification than 16S rRNA gene [14]. Among the reference earthworm species, the closest evolutionary relation of *L. violaceous* for 18S rRNA partial nucleotide sequence was *Eudrilidae *sp, although the taxonomy to the specie level of *Eudrilidae* sp. still remains imprecise. Chang [58] had alleged many incorrect earthworm species sequences in the GenBank which results in misleading conclusions. This however, cannot be adjudged so in our conclusion since the phylograms for *L. violaceous* 18S rRNA and 16S rRNA indicate evolutionary differences between *L.* *violaceous* and *Eudrilidae* sp. 

Although pollutants like metals are known to modify DNA [59] or cause epigenetic responses like methylation and locus variability [21-23], there were no methylations in the specimens sampled in this study. The sampling site is an oil polluted site post incident of oil spill and inferno earlier reported to have heavy metal concentrations [60], it possibly had undergone natural attenuation over the 8yrs [61, 62]. From the earlier report, levels of Zn, Cd, Mn, Ni, V, Pb, Cu and Cr in both polluted and control sites were all below the FEPA standard threshold value [62] hence could be why the metals did not cause epigenetic impact on the 18S rRNA gene of this earthworm species. It could also be due to the fact that the metals monitored do not cause epigenetic impact on earthworm species since none of these metals have been reported to cause methylation of 18SrRNA gene in any available literature. Arsenic is one of the few metals reported to cause epigenetic responses resulting in variation among species of organisms [21]. 
